# Complex Hepatic Abscess in a Child Following Esophageal Procedures: Clinical Insights and Management

**DOI:** 10.7759/cureus.85741

**Published:** 2025-06-10

**Authors:** Jack Hachem, Anthoula Christodoulou, William Hunt, John Manaloor, Javier J Monagas

**Affiliations:** 1 Pediatric Gastroenterology, Baylor College of Medicine, San Antonio, USA; 2 Pediatrics, Baylor College of Medicine, San Antonio, USA; 3 Pediatric Infectious Diseases, Baylor College of Medicine, San Antonio, USA

**Keywords:** eikenella corrodens, esophageal infection, esophageal stents, esophageal stricture, hepatic abscess, prevotella

## Abstract

This case report describes a rare occurrence of a hepatic abscess in a five-year-old girl following esophageal interventions due to caustic ingestion. Persistent strictures led to multiple balloon dilations and serial esophageal stents, after which she developed abdominal pain, fever, and anorexia. Imaging revealed a complex hepatic abscess. Cultures identified *Eikenella corrodens* and *Prevotella oralis*, organisms typically found in the oral cavity, suggesting bacterial translocation due to mucosal disruption from the esophageal procedures. The patient initially received ceftriaxone and metronidazole, showing improvement, but experienced fever recurrence upon switching to ampicillin/sulbactam, necessitating a return to the original antibiotic regimen. The patient eventually recovered with continued treatment, highlighting the risk of serious infections following esophageal interventions in pediatric patients. This case underscores the importance of considering prophylactic antibiotics to prevent such complications, especially in high-risk pediatric patients undergoing repeated interventions. When infectious complications do occur, both antibiotic management and source control are necessary for definitive treatment. Further research is needed to establish guidelines for prophylactic measures in similar clinical scenarios.

## Introduction

Esophageal strictures resulting from caustic ingestions often necessitate endoscopic and surgical interventions. When endoscopic dilatation fails or in cases of long segment strictures, severe caustic injuries, or recurrent strictures, esophageal replacement becomes a critical option. One surgical technique employed is the reversed gastric tube esophagoplasty. This procedure involves isolating a segment of the stomach, which is then fashioned into a tube while preserving its blood supply. This newly formed tube is anastomosed to both the remaining esophagus and stomach, effectively bypassing the damaged or absent esophageal segment. The reversed orientation of the gastric tube aids in maintaining peristalsis and facilitating normal swallowing.

Despite its benefits, stricture formation is a relatively common complication in approximately 30% of esophageal reconstruction surgeries involving gastric tubes [1]. Managing these strictures is challenging and may lead to complications such as perforations, fistulas, and infections. Endoscopic and surgical interventions, such as balloon dilation or gastroplasty, can disrupt the gastroesophageal mucosal barrier, potentially facilitating bacterial translocation into the local venous circulation. There have been documented cases of complicated hepatic abscesses arising from esophageal procedures [2-6]. In this paper, we present a case involving a five-year-old patient who developed a complex hepatic abscess secondary to esophageal interventions. This case highlights the potential complications and challenges associated with treating esophageal strictures in pediatric patients.

## Case presentation

**Figure 1:**
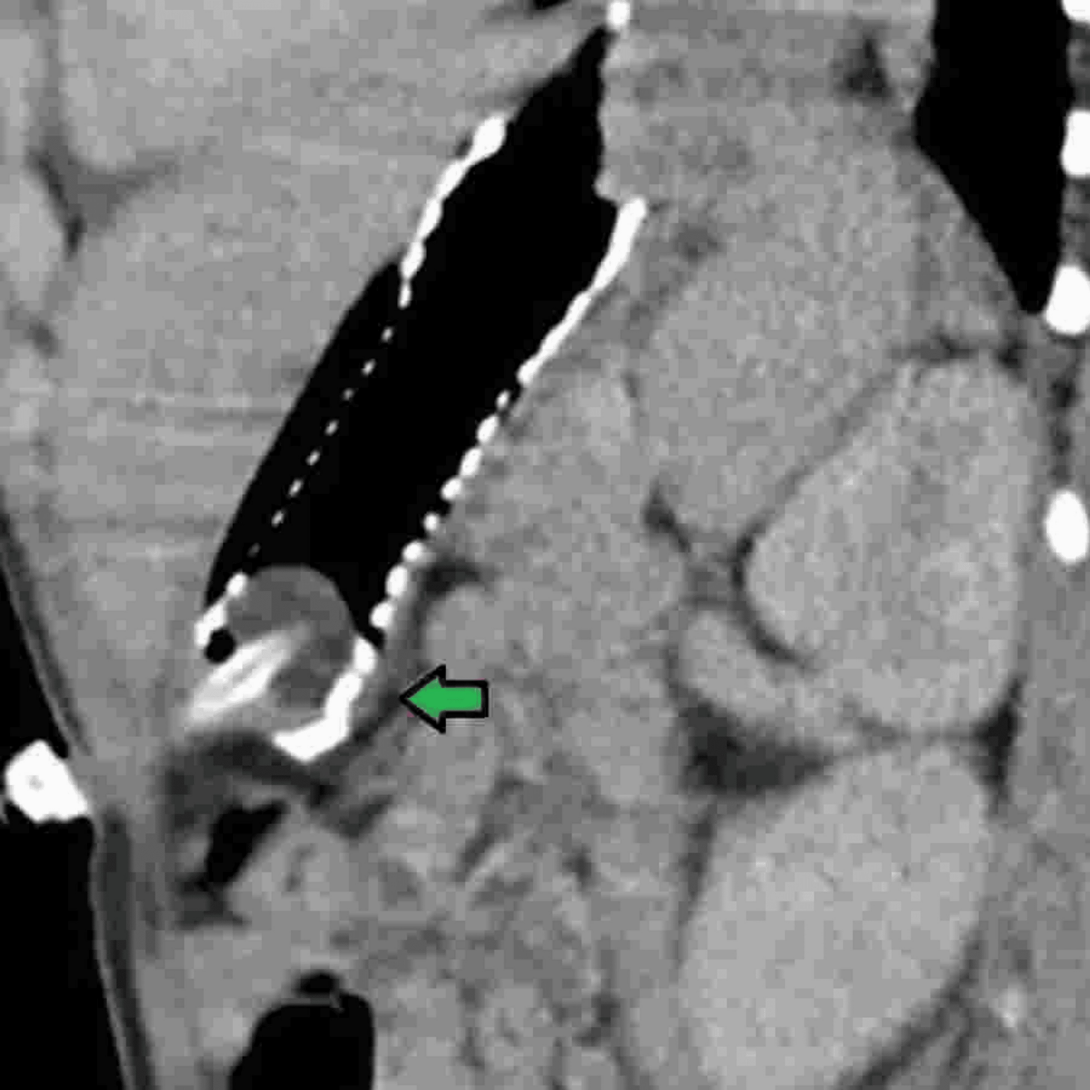
Radiograph image of stent migration to the stomach, with the distal portion wrapped around the gastrostomy balloon (green arrow).

A five-year-old girl with a history of ingestion of a caustic cleaning agent at one year of age underwent a reverse gastric sleeve reconstruction after failing multiple esophageal dilations. Post-surgery, she continued to experience recurrent strictures at the anastomotic site, necessitating further esophageal balloon dilations and ultimately the placement of a fully covered esophageal metal stent. This intervention led to transient improvement, allowing her to tolerate a soft mechanical diet. However, approximately two weeks after the stent placement, she began experiencing progressive abdominal pain, fevers, with a maximum temperature of 102°F, and anorexia with weight loss. She was taken to urgent care and was found to have an elevated white blood cell count (WBC) of 20,000 cells/mm^3^ as well as elevated transaminases, prompting transfer to our emergency department. Upon examination, the patient was febrile, appeared sickly, and was pallorous, with no signs of jaundice or icterus. The abdominal exam was notable for right upper quadrant tenderness and an increased liver span, measuring 2 cm below the costal margin. Additional labs at our hospital showed an elevated CRP at 37.84 mg/dL, an ESR of 93 mm/hr, and a procalcitonin level of 0.68 ng/mL (Table 1). Blood cultures, GI pathogen polymerase chain reaction (PCR) panel, and *Entamoeba histolytica* antigen tests were obtained and later found to be negative. A chest radiograph revealed interval migration of the esophageal stent, with the distal portion wrapped around the gastrostomy balloon (Figure 1).

**Table 1:** Initial laboratory test results upon patient's presentation to the emergency department. PTT: partial thromboplastin time, PT: prothrombin time.

Lab Test	Result	Reference Range
White blood cell (WBC) count	20,000	4,500-11,000 cells/mm^3^
Hemoglobin	8.1	12.5-16.5 g/dL
Platelet count	483,000	150,000-450,000/mm^3^
Sodium	145	136-146 mEq/L
Potassium	3.91	3.5-5 mEq/L
Chloride	101	95-105 mEq/L
Bicarbonate	20	22-28 mEq/L
Albumin	2.7	3.3-4.5 g/dL
C-reactive protein (CRP)	37.8	<0.50 mg/dL
Erythrocyte sedimentation rate (ESR)	>145	0-10 mm/hr
Procalcitonin	0.68	0-0.5 ng/mL
Alanine transaminase (ALT)	100	10-33 U/L
Aspartate aminotransferase (AST)	67	10-33 U/L
Bilirubin total	0.4	0.1-1 mg/dL
PT	14.4	12.3-15.1 sec
PTT	48	24.5-36.1 sec
Lipase	5	8-78 U/L

**Figure 2:**
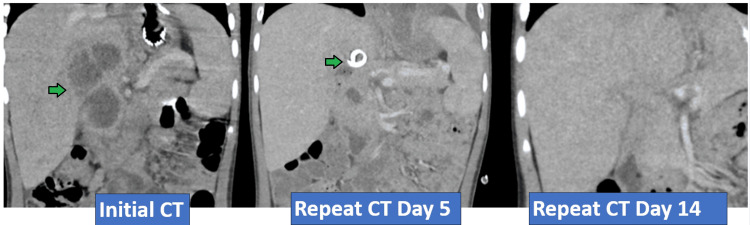
CT depiction of complex septated fluid collection in the hepatic hilum before and after intervention. Green arrow in initial CT and repeat CT day 5 indicates complex septated fluid collection in the hepatic hilum.

Further imaging was obtained with IV and oral contrast computed tomography (CT) of the abdomen/pelvis, revealing a complex septated hepatic abscess at the hilum (4.1 x 3.8 x 4.6 cm). The patient was empirically started on parenteral antibiotics with ceftriaxone and metronidazole. The stent was successfully retrieved via the gastrostomy site. Interventional radiology placed an 8-French Cook percutaneous drain that remained in place for six days. Brown purulent fluid was drained and sent for aerobic and anaerobic cultures. The Gram stain of the fluid showed many polymorphonuclear leukocytes and heavy growth of gram-negative rods, later revealed to be *Eikenella corrodens* and *Prevotella oralis.* 16S Next-Generation Sequencing (NGS) results confirmed abundant *Prevotella loescheii* and moderate *Prevotella oralis* presence. Fungal and acid-fast bacteria (AFB) stains were negative. Repeat labs revealed a decrease in WBC count from 20,000 to 15,300 cells/mm^3^ and CRP down to 15.5 mg/dL after three days of empiric antibiotics with Ceftriaxone and Metronidazole. Based on infectious disease recommendations, we switched to Ampicillin/Sulbactam to narrow the coverage, as it is effective monotherapy for *Eikenella*. However, this resulted in a recurrence of fever and a rebound of CRP to 25.5 mg/dL on day six of antibiotic therapy. The patient was switched back to parenteral ceftriaxone and oral metronidazole. The fever defervesced and repeated labs after eight days of antibiotic therapy improved with a WBC count of 8,400 cells/mm^3^ and a CRP of 11.39 mg/dL. The patient remained afebrile and completed an additional two weeks of parenteral ceftriaxone and PO metronidazole. WBC before discharge was 7,500 cells/mm^3^, and the CRP was 0.24 mg/dL. The patient was sent home on a six-month course of daily enteral antibiotic prophylaxis with amoxicillin-clavulanate. Repeat CT imaging at the end of therapy showed resolution of the abscess (Figure 2).

## Discussion

Endoscopic and surgical procedures, such as endoscopic balloon dilation or gastroplasty, can be traumatic and disrupt the gastroesophageal mucosal barrier, potentially facilitating bacterial translocation into the local venous circulation. The incidence of bacteremia after esophageal reconstruction is up to 56% in the immediate postoperative period and range from 22% to 72% of cases involving repeated esophageal balloon dilation, particularly when the stricture is malignant [7-9]. Complicated infections, including brain abscesses, endocarditis, and meningitis, have been documented [2]. However, only a few cases of complicated hepatic abscesses related to esophageal procedures have been published. Lopez-Tobaruela et al. reported a 55-year-old woman who developed a hepatic abscess approximately two weeks after receiving a biodegradable esophageal stent for an upper esophageal stricture [3]. The mucosal tears from the esophageal dilations likely compromised mucosal integrity, allowing bacteria to enter the bloodstream and subsequently the liver. Similarly, Cirimele et al. described a 31-year-old who developed hepatic abscesses two to three weeks following endoscopic sleeve gastroplasty, and Kessler and Kourtis reported a 53-year-old with an impacted fishbone who developed a hepatic abscess four weeks after the initial insult [4-6]. To our knowledge, there are no pediatric cases for comparison. However, we believe these cases share a common theme of disruption to the gastroesophageal mucosal integrity, allowing oral bacteria to enter the gastroepiploic veins, subsequently seeding into the portal vein and extending to the hepatic hilum, where the bacteria localize into a complicated abscess. A comparison table of the currently published cases is provided in Table 2.

**Table 2:** Published cases of hepatic abscesses in the setting of gastroesophageal mucosal compromise.

Case	Age/Sex	Causes of Mucosal Compromise	Clinical Presentation	Identified Organism	Anti-infective Therapy
Lopez-Tobaruela et al. (2021) [3]	55 y/o F	Multiple bougie/balloon dilations and stent placement	Fever to 102°F. Chest and epigastric pain radiating to the right hypochondrium. Leukocytosis (13,630 cells/µL). Elevated CRP (20.9 mg/dL).	Streptococcus intermedius	Levofloxacin
Cirimele et al. (2023) [4]	31 y/o F	Endoscopic sleeve gastroplasty	Two-day history of fever up to 102°F, epigastric pain two weeks after endoscopic sleeve gastroplasty. Leukocytosis and elevated C-reactive protein levels.	Streptococcus intermedius	Cefotaxime, gentamicin
Kessler and Kourtis (2001) [5]	53 y/o M	Impacted fish bone	Four-week history of abdominal pain and a tender right upper quadrant mass. Leukocytosis (8,100 cells/µL).	Eikenella corrodens	Vancomycin, piperacillin-tazobactam
This study	5 y/o F	Multiple balloon dilations and stent exchange in a neoesophagus	Three-day history of progressive abdominal pain, fevers up to 102°F, anorexia, and weight loss two weeks post-esophageal stent placement. Leukocytosis (20,000 cells/µL), elevated procalcitonin (0.69 mg/dL), and elevated C-reactive protein levels (37.8 mg/dL).	*Prevotella oralis*, *Eikenella corrodens*	Ceftriaxone, metronidazole, amoxicillin-clavulanate

*Eikenella corrodens* is commonly part of the oral flora and is often associated with human bites. There is very little published literature implicating *Eikenella *in hepatic abscess infections. In the case reported by Kessler and Kourtis, it was presumed that the *Eikenella *organism was acquired from the patient's oral cavity, while he was eating fish [5]. Similarly, in our patient, during the initial endoscopic insertion, the endoscope could acquire *Eikenella corrodens* when it contacts the oral mucosa before entering the esophagus. *Eikenella *could then presumably be introduced through mucosal tears during dilation or stent therapy. This mechanism underscores the potential vulnerability of patients with invasive esophageal procedures.

*Prevotella* is also an integral part of the oral flora. *Prevotella oralis* is frequently found in subgingival plaque and is associated with periodontal disease, including gingivitis, periodontitis, and dentoalveolar abscesses [10]. It can also extend beyond its primary habitat in the oral cavity. *Prevotella loescheii* has been known to cause infections in prosthetic joints, such as total hip arthroplasty, likely due to hematogenous spread from oral sources. *Prevotella oralis* has been identified in systemic infections, notably in cases of bacteremia of hepatic origin, where it has been isolated from hepatic abscess drainage [11].

The onset of liver infection seems to occur several weeks following compromise to the mucosal barrier. Currently, no standard practice exists for administering prophylactic antibiotics to patients undergoing endoscopic therapies or surgeries that compromise the mucosal barrier. However, antibiotic prophylaxis in patients undergoing repeated endoscopic therapies, such as dilation, stent placement, or stent replacement, particularly those with esophageal reconstruction or who are immunocompromised, may be beneficial in managing these patients. This approach would be similar to antibiotic prophylaxis use in patients undergoing dental procedures that are high risk for bacteremia [12]. In general, an antibiotic effective against anaerobic organisms, such as Augmentin, should be considered. A second agent may be necessary to ensure broader spectrum coverage for all potential esophageal pathogens. It is advisable to consult with a clinical pharmacist, as clinical data on this topic is limited.

## Conclusions

Our case is particularly unique due to the rare complication and the unusual organisms involved. Notably, no pediatric cases have been reported, nor have any cases involving *Prevotella oralis*. The implications of this case highlight the importance of considering antibiotic prophylaxis for patients undergoing frequent esophageal interventions. Prophylactic antibiotics could significantly reduce the risk of bacterial translocation and subsequent abscess formation. This approach is especially pertinent for pediatric patients with complex esophageal reconstructions, who are at increased risk for such complications. Ensuring appropriate antibiotic coverage during and after these procedures could help mitigate severe infections and improve patient outcomes.

While the risk of infection following endoscopic procedures is not universally acknowledged, the potential for serious complications such as hepatic abscess formation warrants consideration of prophylactic measures in high-risk patients. Further research and clinical guidelines are needed to establish protocols that can effectively prevent these rare but significant complications.
